# Load Measurement on Foundations of Rockfall Protection Systems

**DOI:** 10.3390/s16020174

**Published:** 2016-01-29

**Authors:** Axel Volkwein, Peter Kummer, Hueseyin Bitnel, Lorenzo Campana

**Affiliations:** 1 WSL Swiss Federal Institute for Forest, Snow and Landscape Research, Zürcherstr. 111, 8903 Birmensdorf, Switzerland; peter.kummer9@gmail.com; 2 Geobrugg AG, Aachstr. 11, 8590 Romanshorn, Switzerland; Hueseyin.Bitnel@geobrugg.com; 3 Institute of Forensic Medicine, Bühlstrasse 20, 3012 Bern, Switzerland; Lorenzo.Campana@irm.unibe.ch

**Keywords:** rockfall, protection systems, net barrier, testing, post, foundation

## Abstract

Rockfall protection barriers are connected to the ground using steel cables fixed with anchors and foundations for the steel posts. It is common practice to measure the forces in the cables, while to date measurements of forces in the foundations have been inadequately resolved. An overview is presented of existing methods to measure the loads on the post foundations of rockfall protection barriers. Addressing some of the inadequacies of existing approaches, a novel sensor unit is presented that is able to capture the forces acting on post foundations in all six degrees of freedom. The sensor unit consists of four triaxial force sensors placed between two steel plates. To correctly convert the measurements into the directional forces acting on the foundation a special *in-situ* calibration procedure is proposed that delivers a corresponding conversion matrix.

## 1. Introduction

**Figure 1:**
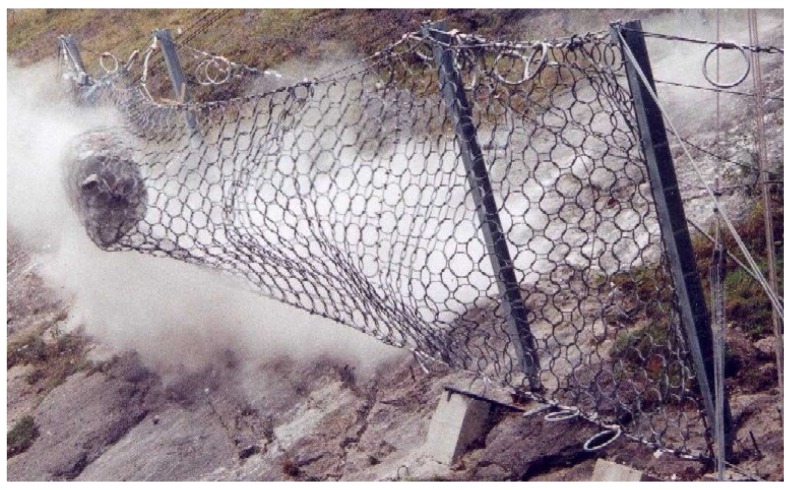
Example of flexible rockfall protection barrier in action (source: Fatzer).

**Figure 2:**
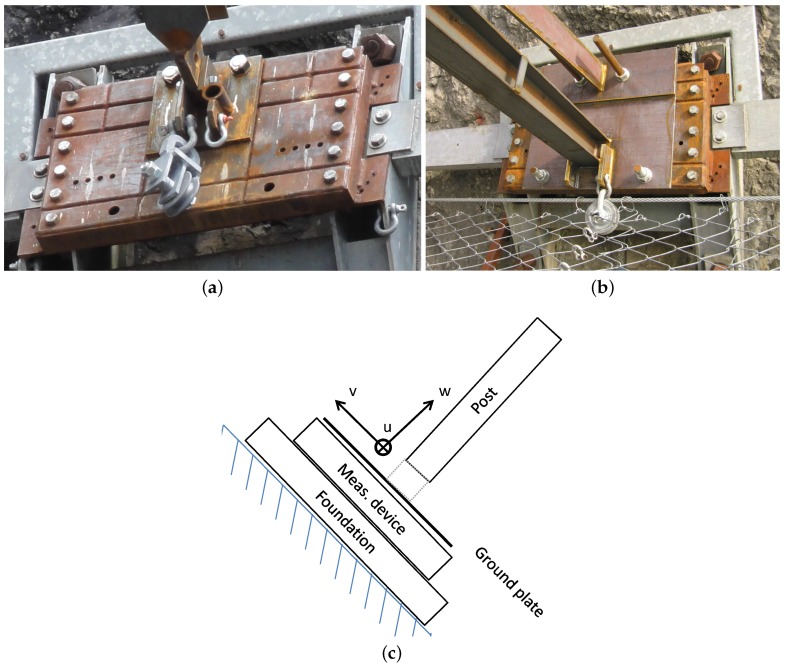
Example of steel posts used in rockfall protection barriers (**a**) with hinged support, upslope ropes supporting the post head and (**b**) with fully clamped support; (**c**) Sketch with nomenclature of component parts at the post base.

Fence-like steel structures are often used to protect against rockfall. The so-called flexible rockfall protection barriers consist of a main steel wire net that is spanned by steel cables and posts (see Figure 1). The steel posts usually have either a hinged or clamped support at the base (see Figure 2a,b). When testing rockfall protection barriers for approval according to [1,2], it is stipulated that measurements of the loads in the barrier’s ropes are carried out to provide clients with the necessary loads for the corresponding anchorages. However, loads experienced by post support plates and underlying foundations [3] are not a testing requirement. This is currently the case, because up to now it was not feasible to measure loads exceeding 1 MN normal force and 300 kN lateral force, and thus guidelines on testing do not define the required measurements. For hinged posts such forces are usually estimated and the corresponding foundations are designed with a large factor of safety. The estimations are based on the forces measured in the ropes that are attached to post heads and at the post bases or in adjacent ground plates. This approach may be sufficient to account for the main pressure loading that is transferred to the ground by hinge supported posts. However, it does not account for the high tensile forces sustained by post foundations and anchorages commonly used for clamped posts.

Another reason for more detailed measurements is the increasing number of numerical simulations of such flexible protection systems as described in [4,5,6,7]. The results from the field tests are the only possibility of calibrating and validating the simulation procedures. Hence, the more data retrieved during testing the better the performance of the numerical simulation.

In this respect the corresponding measurements are invaluable. For this purpose, various approaches exist that are described in Section 3 all measuring single or just some of the force/torque loads acting on the foundation.

Addressing these requirements, we propose a novel measurement system permitting loads on the post support in all six degrees of freedom (DOF) to be determined, *i.e.*, forces and torque loads in three orthogonal directions. We present the complete structural and metrological setup, the installation and testing experience in the field in addition to data capture and processing. Given the fixed installation in the field, we also propose an *in situ* calibration procedure, which allows a regular surveillance of the measurement system and fulfils the calibration requirements given in [1] for the approval tests of rockfall protection systems.

## 2. Boundary Conditions for a 6-DOF Measurement System

Different boundary conditions must be considered to setup a suitable measuring system that detects loads acting on post foundations and support plates of a rockfall protection barrier system. The first decision to be made is the choice of the load directions that should be measured. As shown above, the possibilities range from single direction and pressure only measurements to multi-directional measurements including bending moments / torques. If only specific directions are to be investigated, their actual orientation is influenced by different factors such as the post inclination, the orientation of the foundation’s surface or the inclination of the slope on which the the barrier is mounted. These parameters can vary for each installation at the testing site or for the field conditions in which the protection systems are erected. Furthermore, the orientations must be identified for the design of each different system post. For example, a hinged support only transfers forces into the foundation whereas a clamped supported post also imposes torque. It is questionable as to whether torque loading should be measured because the standard field installation is implemented by means of a pair of forces with a foundation carrying the pressure loads with drilled anchors under tension. Basically, the chosen measurement is influenced by both the technical and economic capacity, in addition to the installation geometry and the required measurements. Given the advances made in rockfall protection barriers [8] in our assessment a post base measurement device should be capable of detecting loads in all possible directions, *i.e.*, six degrees of freedom. A setup of this kind will ensure comprehensive measurements of post foundation forces to accommodate any further advances in the design of rockfall protection barriers.

Essential to the design of a suitable measuring device are the expected loads. In this case, we estimated the post forces with results from tests on typical rockfall protection systems, in which the loads in different steel ropes (as shown in Figure 1) have been measured. Hereby it is assumed that these measured forces and loads can be used to estimate the loads in the steel post and its foundation. Based on these assumptions, we defined a minimum load of 1000 kN orthogonal to the ground plate and 300kN parallel to the ground plate. These magnitudes excluded many measurement systems that were available on the market and therefore required the development of a specially adapted system.

**Figure 3:**
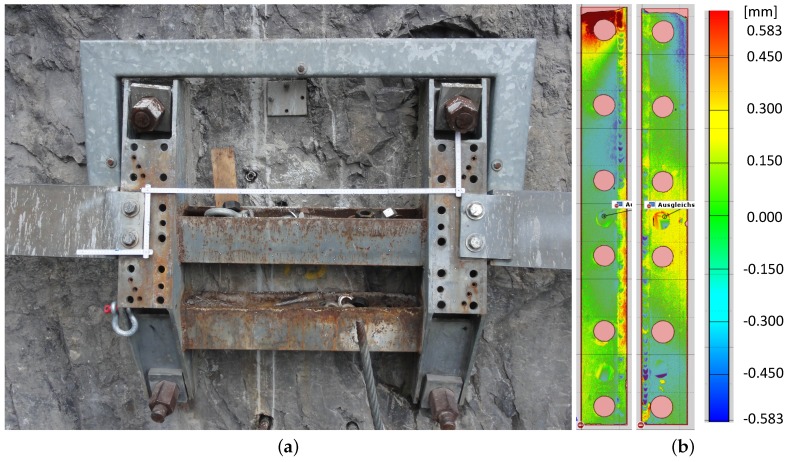
(**a**) Two strip foundations mounted on the rock wall; (**b**) Orthogonal deviation of the surface of the two strip foundations from a common plane, determined using a high precision laser scanner.

Necessary adaptations are also related to the geometrical conditions on site. In our case, the measuring device should replace a steel plate of dimensions 900×590×100 mm on top of which the ground-plate for the steel post is attached (see Figure 2c). The plate must fit to the bolt pattern of the strip foundations (Figure 3) and of the original steel plate which provides four anchor holes to attach the ground plate (Figure 2a,b). Apart from the external fixations the measuring device itself can be slightly larger than the original steel plate. Furthermore, the local situation allows a slight increase of the barrier height due to the device.

The sensor unit is installed at the test site on a rock wall, which requires that throughout the year it is resistant to humidity, precipitation and temperature especially when exposed to the sun and during winter time.

## 3. Options for a Post Plate Measuring Device

**Figure 4:**
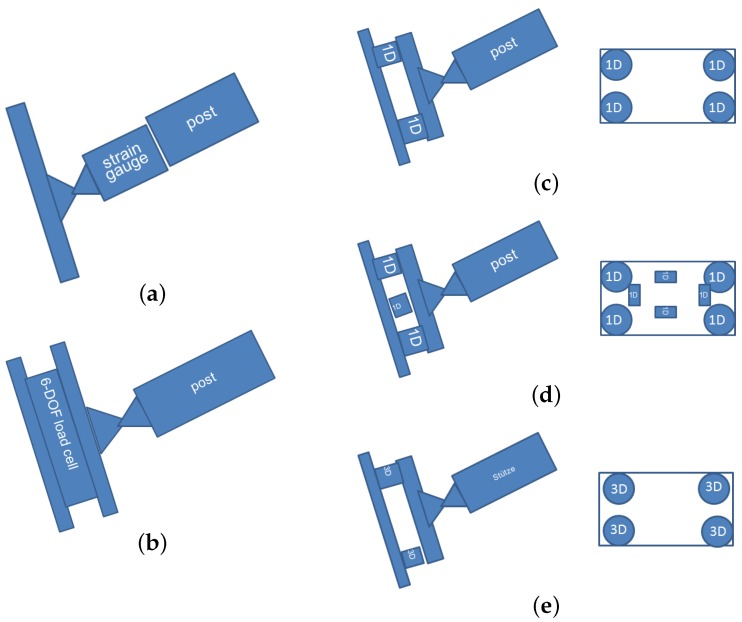
Different possible setups to measure 6-DOF at a post base. (**a**) Instrumented steel post; (**b**) single 6-DOF load cell; (**c**) four one-dimensional load cells and (**d**) with additional orthogonal load cells for plate parallel forces; (**e**) four triaxial load sensors.

There are a number of possible setups which can deliver a reliable post base measurement, each having advantages and disadvantages. Figure 4 presents the ideas that are discussed in the following. A special preparation of a steel post with strain gauges (Figure 4a, applied by e.g., [9]) follows a different approach and directly delivers the loads within the post. However, every new post has to be equipped with new sensors. This requires special attention to the calibration of the measurement, especially for bending and shear loads. Furthermore, if only the steel post is measured, additional loads acting on the ground plate and foundation are neglected such as ropes that pass through the post base plate.

The most elegant and easy solution would be to use a single multi-component transducer (Figure 4b). However, research into ready-made sensors reveals that the sensors are limited to loads that are much smaller than required for the intended application. On the other hand, there are sensor manufacturers capable of developing such a sensor (e.g., [10]). In this case, it must be ensured that the manufacturer is able to evaluate and to calibrate the sensor for the full sensor range prior to delivery, which would require a multi-component testing installation to also quantify the interference of single load directions. The only disadvantage of this option are the potentially high costs involved in the development of such a device.

The proposed setup of Figure 4c would use three or four 1D tension/pressure sensors between two steel plates. Such sensors have to be insensitive to transverse loads (e.g., HBM U5 [11]). Both versions with three or four load cells allow torques around the x- and y-axes according to Figure 2c to be determined with the difference of static determinacy or indeterminacy. However, the loads parallel to the plate surfaces and the torsion around the z-axis cannot be determined with this method. In our case, the sensors described in [11] were shown to require too much space between the steel plates, making the fixation of the ground plate problematic.

Adapting the aforementioned solution to measure loads parallel to the plate, the setup can be equipped with further plate-parallel sensors (Figure 4d). Depending on the available sensors the construction remains small in height. The structural implementation also provides a mechanical decoupling of the single sensors. However, the entire setup is complex and also reacts with high sensitivity to minimal geometrical changes.

Figure 4e shows a setup with four triaxial load cells fix-mounted between two steel plates. With this setup all six degrees of freedom can be investigated, *i.e.*, the forces normal and parallel to the plates and the torques around these axes. In total twelve measurement channels exist. This results in a six-fold indeterminacy regarding the only six unknowns and requires corresponding mathematical handling of the raw data obtained as described, for example, in [12,13]. However, the same indeterminacy allows an additional evaluation of the single measurement channels detecting measurement errors. An additional drawback of this approach is the inevitable force shunt between the single load cells and the single measurement channels, which results from their coupling with the rigid connecting steel plates. In this case a specially adapted calibration procedure has to be established as mentioned in [14].

## 4. Existing Post Base Measurement Approaches

**Figure 5:**
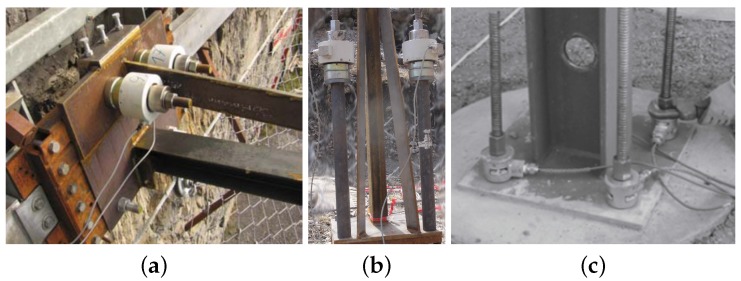
(**a**) Pressure load cells with load distribution ring and ball scraper to measure tension forces on anchors directly between ground plate and anchor nut and (**b**) with pipe elongations in case the space on the ground plate is too small for the load cells; (**c**) shows a setup with four load cells (source: [16]).

Different approaches have been developed to provide data of support loads in clamped or hinged posts of rockfall barriers. Probably the simplest approach is to measure only the relevant tension loads using pressure cells placed between the support base plate and the anchor nuts, which fix the plate to the ground (Figure 5a). Several accredited rockfall protection barrier systems have been approved using this approach (listed in [15]). Where space is constricted on the ground plate steel pipes enable the load cells to be placed away from the ground plate (Figure 5b). Due to the pretension applied to the anchors this setup remains stable. If pressures acting on the foundation beneath the ground plate are to be measured, additional load cells have to be installed between them. Alternatively, the pretension of the anchor-load-cell combination is high enough to fully cover a load reduction during pressure loading [16]. The load cells HBM C6A shown in Figure 5a work with a 10 kHz sample rate, while 2–4 kHz is usually sufficient for typical applications with rockfall protection barriers, for which the braking process lasts about 0.2 s. For expected loads of up to maximum 500 kN the measurement range of the load cells was chosen to be 1 MN. This is selected according to the manufacturer’s recommendations, whereby loads should not exceed 30%–50% of the load cell measurement range due to the dynamic nature of the quickly changing loads in addition to the high sampling rate. The selected load cells react sensitively to uneven loading along load transmitting surfaces and therefore need an additional set of load distribution rings and a ball scraper. [16] apply pressure cells to four anchors attached to a single ground plate (Figure 5c). With sufficient pretension this setup also allows torque loads around the axes parallel to the ground to be obtained.

**Figure 6:**
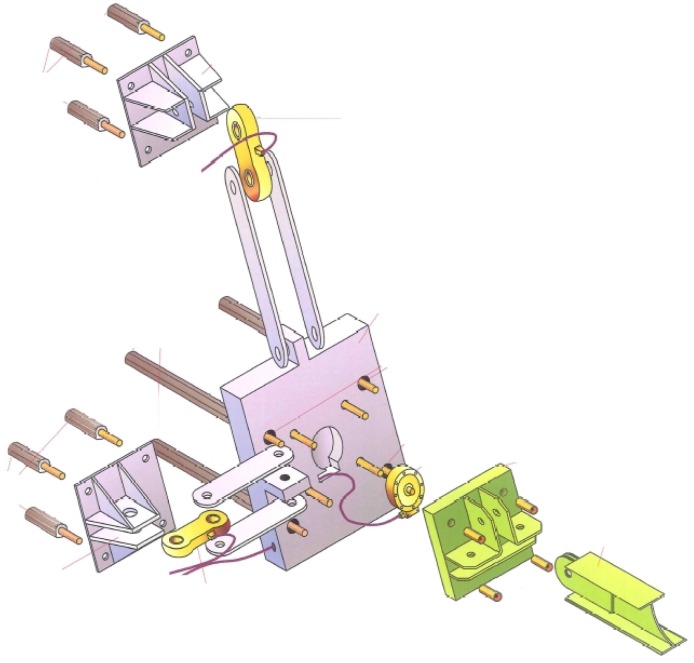
Patented measurement system for loads in the case of hinged steel posts and the ground plate orthogonal and parallel to the ground (source: [3]).

The patented [17] setup described in [18] and shown in Figure 6 can be applied to hinged steel post supports to measure the pressure load orthogonal to the ground in combination with ground parallel loads. Three different load cells of this design are combined kinematically independent of each other.

**Figure 7:**
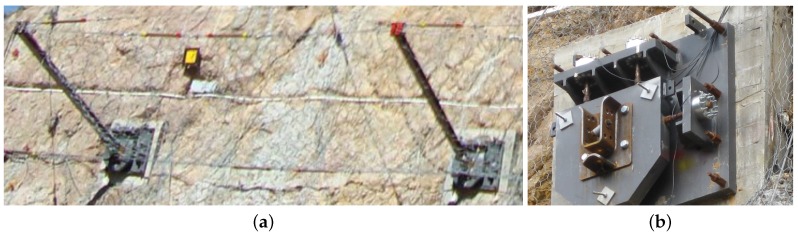
Post base measurement system (**a**) with installed posts at a test site and (**b**) in detail showing three pressure sensors orthogonal to the rock face and three two-way sensor installations parallel to the rock face (source: Igor Paramassi S.p.a.).

The post base measurement system shown in Figure 7 consists of six load sensors measuring pressure loads orthogonal and parallel to the ground. The system setup is statically determinate in orthogonal and parallel directions each using three sensors. If tension-pressure sensors are used in the plate-orthogonal direction and correspondingly connected to the top steel plate then it is also possible to deduce torque loads around the two parallel axes from the measurements, in addition to torque around the orthogonal axis analogous to the arrangement of the ground parallel sensors.

## 5. Description of the Sensor Unit

It was our intention to run a compact measuring device with a reduced need for maintenance. The measuring device is installed 15 m above ground on a rock wall. Once installed the system remains mounted throughout the year, summer and winter. Regular mounting and demounting would be too laborious. In addition, to ensure that maintenance remains low, the number of components of the sensor unit should be kept to a possible minimum. However, the development of a suitable single sensor measuring 6 DOF was too costly and ready-made sensors suitable for the expected load level were not available at that time. Therefore, we decided to set up a sensor system consisting of four triaxial force sensors (Figure 4e) based on the possibilities and options discussed above. The system is robust for permanent outdoor use, requires limited amount of steel works and provides the detection of all six degrees of freedom.

### 5.1. Sensor Hardware

**Figure 8:**
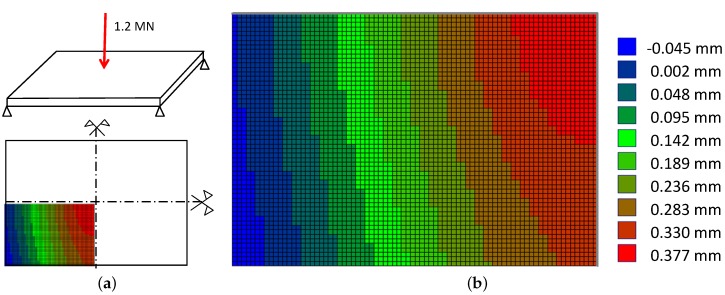
Doubly symmetric FE simulation of the top steel plate: (**a**) A pressure of 1.2 MN simulates the load of a steel post and loads the center of a rectangular steel plate; (**b**) Deflection of one quarter of the plate in the load direction.

The four sensors should measure in three directions, each fulfilling at least IP67, and be robust enough to withstand overloading or unwanted distortion, in this case due to bending of the two steel plates. Suitable sensors can be found on the market from different manufacturers. The final decision was to use four GTM RF300 sensors [19] with additional channels for loads orthonormal to the main axis. The cells are placed between two steel plates with an area of $850\times 75\;{\mathrm{mm}}^{2}$ on which the steel post can be mounted. An 80 mm thick steel plate was selected to ensure that their maximum deformation did not exceed the maximum allowable elastic deformations of the load cells (see Figure 8 for an example of a Finite Element simulation of the sheet deflections). Further, it has to be checked that the load measurement itself is not falsified during a dynamic measurement due to the inertia of the upper steel plate with a mass of roughly m=400 kg: If we assume a maximum deflection of 0.04 cm (see Figure 8) all over the steel plate and that this deflection builds up within about 0.05 s. We obtain an inertial load of $F=m\cdot a=400\;\mathrm{kg}\cdot 0.0004\;m/{\left(0.05\;s\right)}^{2}=64$ N. As this magnitude is small we assume that inertial effects may be neglected.

Four holes were drilled through the top plate to enable fixation of the steel post. Twelve holes are available in the bottom plate to fix it to the foundation. The connection to the foundation has not been equipped with additional shear bearing elements because the pretension of the twelve fixation bolts renders such loads negligible. Each load cell is connected to the corresponding steel plate using twelve M20 screws. Threads are driven into each hole drilled in the steel plate. On each plate two additional 12 mm holes house center pins for a precise positioning of the load cells. Two additionally threaded holes on the top plate provide attachment points for O-rings to facilitate transport and mounting of the sensor unit. An additional sensor unit housing provides protection from direct precipitation and is fixed to the threaded holes of the O-rings.

When mounting four load cells between two steel plates, stresses due to the static indeterminacy are inevitable because the steel plates cannot be manufactured to fulfil an out-of-plane accuracy of less than 0.1mm. To reduce these stresses four additional 0.025 mm steel film washers were placed between two of the load cells and the steel plate.

**Figure 9:**
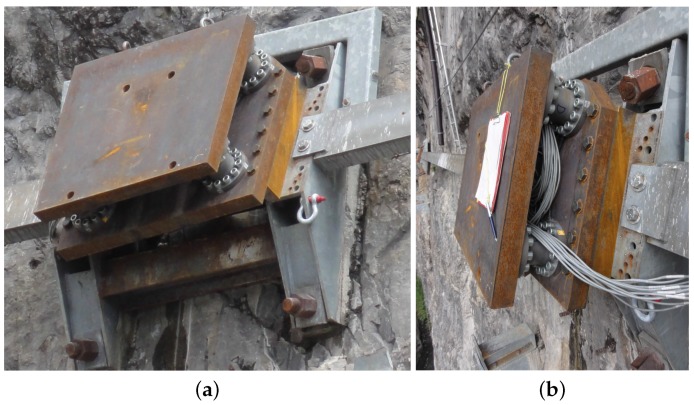
(**a**) Oblique and (**b**) lateral view on mounted sensor unit. Additional steel alignment sheets can be seen between the lower steel plate and the galvanized foundation.

Special care was taken for the installation of the sensor unit to the two strip foundations, which are shown in Figure 3b. The two strips are not coplanar which requires a corresponding alignment. In a first attempt we mounted the sensor unit with several alignment sheets between the sensor unit and the strip foundations. However, this procedure is rather time-consuming, has a limited repeatability in the case of remounting and does not provide the intended laminar contact surface between the steel plate and the strip foundations. To enable precision design of the alignment sheets a high-resolution three-dimensional scan of the foundation strips was performed. The scan was performed using a GOM ATOS III (Rev. 01) [20] with a resolution of 0.28 mm and a precision of 0.01 mm. Before the scan, the strip foundations and the surrounding rock face were equipped with small reference marks to facilitate merging of the two scans to obtain the topography of the strip foundation. Figure 3b has been produced with the software analysis tool *GOM Inspect* [21] and shows the deviation of the strip foundations from a common reference plane. The resulting foundation topography gives the negative profile of the two steel alignment sheets to be milled. However, it was not possible to process the scan data to be compatible with the milling device, either as point cloud or as triangulated data. Therefore, we decided to define a single plane for each steel sheet that best fits the underlying foundation topography with a maximum gap of 0.58 mm (see also Figure 9).

The thicknesses of the alignment sheets were also adapted for the orientation of the steel post to be orthogonal to the installation line of the rockfall protection barrier.

The sensor unit is mounted by first placing two steel bolts within the foundations to which the two alignment sheets are hung. The sensor unit is then hung on these bolts and the remaining ten bolts are installed and tightened.

**Figure 10:**
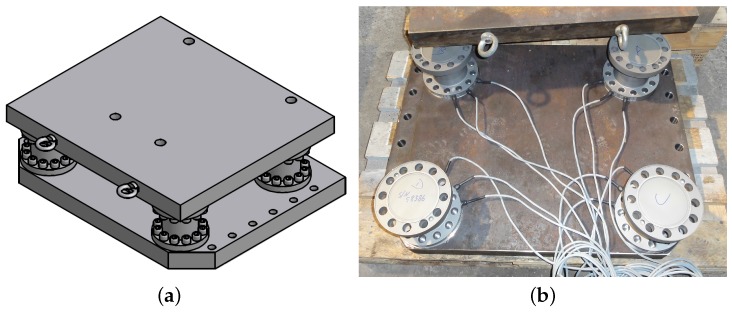
(**a**) Sensor unit consisting of two 80 mm steel plates with four triaxial force sensors mounted between them; (**b**) Arrangement of sensors on steel plate with measuring leads directed inwards.

### 5.2. Measuring Hardware

The four load cells each provide three fully bridged measuring circuits for their local channels in the respective load directions *x*, *y* and *z*. The resulting twelve signal wires of the load cells are then distributed into three extension cords bundled for each direction *x*, *y* and *z* and sent to three signal amplifiers NI9237 [22]. The amplifiers are located in a USB-based data acquisition unit NIcDAQ-9174 [23] which in turn is controlled by the LabVIEW [24] program on a PC. The typical sampling rate of the unit for our application is 2 kHz. Measurements usually last only a couple of seconds during a rockfall experiment, e.g., according to [1,2]. Measurement is automatically initiated with an external trigger signal and additionally captures pre-triggered data about 1 s before the impact of the rock block on the rockfall protection barrier.

### 5.3. Data Processing

The measured data are stored in a single text file with each column containing measurements from each channel *x*, *y*, *z* of each load cell *A*, *B*, *C*, *D*. The data are given in Millivolt per Volt (mV/V). If only dynamic load changes during the rockfall experiment are of interest, we first offset all measurement values of a channel by the average value during the first half second during which the protection barrier is still unloaded.

For each single time step the values are now saved in a vector $R_{\mathrm{raw}}$ (see Equation (1)). For each load cell *A*, *B*, *C*, *D* a calibration document exists from the manufacturer that also contains a 3×3 decoupling matrix K. This matrix allows the removal of signal noise/cross sensitivity generated in two channels caused by the influence the third channel of the load cell. The vector $R_{\mathrm{raw}}$ is therefore converted to $R_{\mathrm{uncoupled}}$ using the decoupling matrices $K_{A}$, $K_{B}$, $K_{C}$, $K_{D}$ by

(1) $$R_{\mathrm{uncoupled}}=\left[\begin{array}{c} a_{x}\\ a_{y}\\ a_{z}\\ b_{x}\\ b_{y}\\ b_{z}\\ c_{x}\\ c_{y}\\ c_{z}\\ d_{x}\\ d_{y}\\ d_{z} \end{array}\right]={\left[\begin{array}{cccc} K_{A}^{T} & 0 & 0 & 0\\ 0 & K_{B}^{T} & 0 & 0\\ 0 & 0 & K_{C}^{T} & 0\\ 0 & 0 & 0 & K_{D}^{T} \end{array}\right]}_{12\times 12}\cdot R_{\mathrm{raw}}\;\mathrm{with}\;R_{\mathrm{raw}}=\left[\begin{array}{c} A_{x,\mathrm{measured}}\\ A_{y,\mathrm{measured}}\\ A_{z,\mathrm{measured}}\\ B_{x,\mathrm{measured}}\\ B_{y,\mathrm{measured}}\\ B_{z,\mathrm{measured}}\\ C_{x,\mathrm{measured}}\\ C_{y,\mathrm{measured}}\\ C_{z,\mathrm{measured}}\\ D_{x,\mathrm{measured}}\\ D_{y,\mathrm{measured}}\\ D_{z,\mathrm{measured}} \end{array}\right]$$

with the dot-operator “·” between two matrices in a normal matrix multiplication.

**Figure 11:**
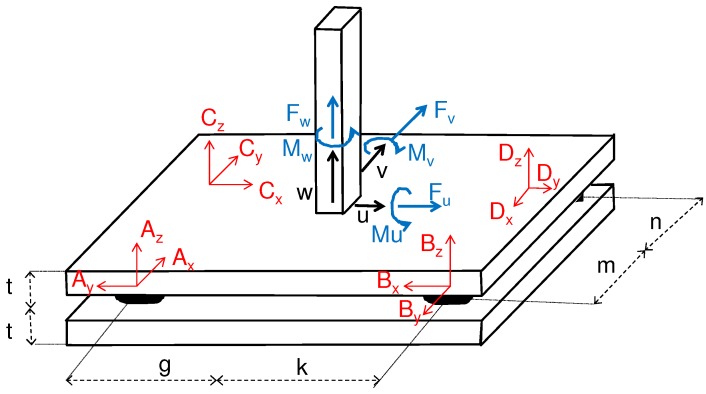
Orientation and position of local coordinate systems *x*, *y*, *z* of single load cells with respect to the chosen global coordinate system *u*, *v*, *w*. Also shown are the directions of the internal torques $M_{u}$, $M_{v}$, $M_{w}$ and forces $F_{u}$, $F_{v}$, $F_{w}$ between the steel post and the measurement plate.

The four sensors *A*, *B*, *C*, *D* are installed close to the corners of the steel plate in such a way that their signal wires are directed to the center of the plate (see Figure 10). Due to this their local coordinate systems *x*, *y*, *z* are rotated with respect to the global coordinate system *u*, *v*, *w* (see Figure 11) by

(2) $$R_{\text{rotated}}=\left[\begin{array}{c} A_{u}\\ A_{v}\\ A_{w}\\ B_{u}\\ B_{v}\\ B_{w}\\ C_{u}\\ C_{v}\\ C_{w}\\ D_{u}\\ D_{v}\\ D_{w} \end{array}\right]={\left[\begin{array}{cccc} \begin{array}{ccc} 0 & -1 & 0\\ +1 & 0 & 0\\ 0 & 0 & +1 \end{array} & & & 0\\ & \begin{array}{ccc} -1 & 0 & 0\\ 0 & -1 & 0\\ 0 & 0 & +1 \end{array} & & \\ & & \begin{array}{ccc} +1 & 0 & 0\\ 0 & +1 & 0\\ 0 & 0 & +1 \end{array} & \\ 0 & & & \begin{array}{ccc} 0 & +1 & 0\\ -1 & 0 & 0\\ 0 & 0 & +1 \end{array} \end{array}\right]}_{12\times 12}\cdot R_{\mathrm{uncoupled}}$$

The actions on the steel plate in all six directions can now be computed in relation to a chosen position between the load cells *A*, *B*, *C*, *D* described by the distances *m*, *n*, *g*, *k* of Figure 11. It also has to be considered that the four force sensors measure with respect to a plane between the two main steel plates at a distance *t* (see Figure 11) below the top surface. This influences the torques acting around axes *u* and *v* when computed for the top steel surface. Finally, the torques $M_{u}$, $M_{v}$, $M_{w}$ and forces $F_{u}$, $F_{v}$, $F_{w}$ with regard to the global coordinate axes *u*, *v*, *w* are then determined by

(3) $$\left[\begin{array}{c} M\\ F \end{array}\right]=\left[\begin{array}{c} M_{u}\\ M_{v}\\ M_{w}\\ F_{u}\\ F_{v}\\ F_{w} \end{array}\right]=\left[\begin{array}{cccccccccccc} 0 & +t & +m & 0 & +t & +m & 0 & +t & -n & 0 & +t & -n\\ -t & 0 & -g & -t & 0 & +k & -t & 0 & -g & -t & 0 & k\\ -m & +g & 0 & -m & -k & 0 & n & g & 0 & n & -k & 0\\ -1 & 0 & 0 & -1 & 0 & 0 & -1 & 0 & 0 & -1 & 0 & 0\\ 0 & -1 & 0 & 0 & -1 & 0 & 0 & -1 & 0 & 0 & -1 & 0\\ 0 & 0 & -1 & 0 & 0 & -1 & 0 & 0 & -1 & 0 & 0 & -1 \end{array}\right]\cdot R_{rotated}$$

The aforementioned operations must now be performed for each time step of the measurement in order to provide the actions on the foundation over time. Of course, the above operations shown in Equations (1)–(3) can be reduced using a single conversion matrix K,

(4) $${\left[\begin{array}{c} M\\ F \end{array}\right]}_{6\times 1}=K_{6\times 12}\cdot R_{\mathrm{raw}}$$

**Figure 12:**
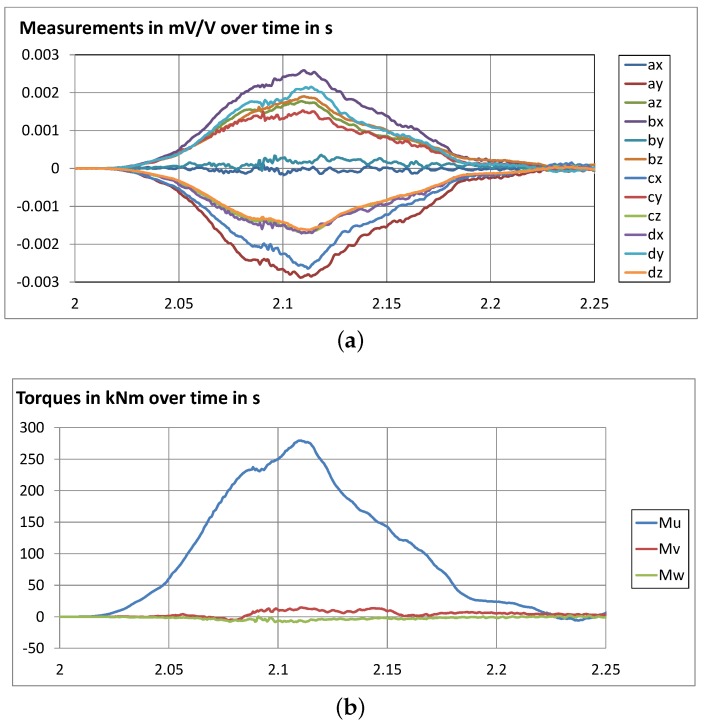

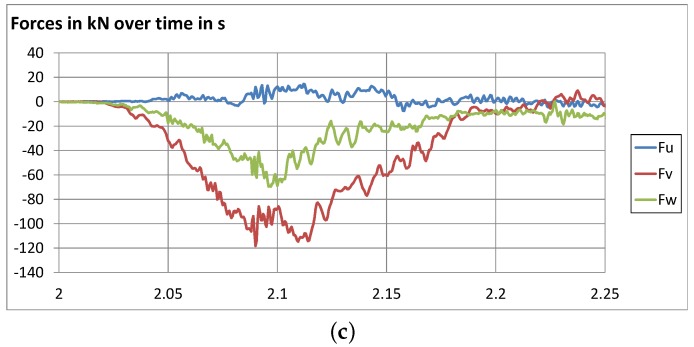
Test of a rockfall protection barrier system with clamped posts: (**a**) measurement; (**b**,**c**) resulting actions on foundation (torques+forces).

As an example, Figure 12 shows the measurement of a dynamic test of a rockfall protection system with clamped posts and the resulting actions, *i.e.*, torques and forces, on the foundation.

## 6. Measurement Validation

In this section, we propose a procedure to validate the measurements and the post-measurement data processed directly in the field. The usual way to initially and regularly check or calibrate such a sensor unit would be an installation in a special multi-axial testing device. However, such devices for the load range of the sensor unit are rare. Furthermore, mounting and remounting of the sensor unit with all the appending 70 m long measuring leads is extremely laborious. The *in situ* procedure has the further advantage that the performance of the sensor unit is studied exactly as it would perform during a rockfall test, *i.e.*, mounted on the foundation in the field including possible special geometrical requirements.

**Figure 13:**
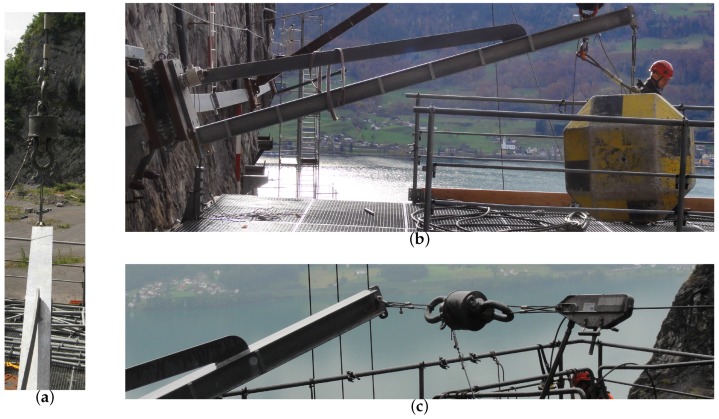
Static loading of a clamped steel post (**a**) vertically upwards; (**b**) vertically downwards; (**c**) sideways.

To validate the measurements, we mounted a roughly 4 m long clamped steel post (see Figure 2b) to the top surface of the sensor unit. At the post head we sequentially applied different forces. Vertically upward using a crane (Figure 13a), vertically downward using a concrete block (Figure 13b) and sideways using a cable winch (Figure 13c). All loads were applied slowly, *i.e.*, quasi-statically, and held constant for a few minutes. The self-weight of the steel post was neglected because only the load changes during each load case are relevant. With the post mounted before testing, we assume a linear behavior of the load cells. Thermal processes due to warming by the sun are neglected because on the test date there was a maximum change of temperature of less than 5 °C and the sun was not shining.

The magnitude of the forces applied were measured using a calibrated 500 kN load cell and sampled every second, or with a manually read 200 kN dynamometer (downward direction). In parallel, the loads measured by the sensor unit are sampled every second. To provide the possibility for an additional check we also mounted two pressure sensors (see Figure 5a) on two anchors of the post with measurements also taken every second.

The points of load application and the exact load directions were determined using a total station (Leica Nova MS50 [25]). These measurements were converted to the coordinate system *u*, *v*, *w* resulting in a position vector r and a load vector S. The corresponding torque T is determined using the vector cross product:

(5) $$T_{3\times 1}=r\times S\;\mathrm{with}\;r=\left[\begin{array}{c} r_{u}\\ r_{v}\\ r_{w} \end{array}\right]\;\mathrm{and}\;S=\left[\begin{array}{c} S_{u}\\ S_{v}\\ S_{w} \end{array}\right]$$

To now check the quality of the sensor unit’s measurements, its derived actions on the foundation can be compared with the loads applied. Table 1 shows a corresponding comparison for the load cases described above. The results show that the numerical signal conversion works in principle. Although this was a first attempt to check the validity of the measurements, discrepancies between the measurement and the applied load are large in some cases (see Table 1). We identified the following issues as possible sources for such mismatches and propose the following improvements for future testing:

- The reference load cell has a weight of 60 kg, which influences the determination of the applied torques T and forces S. For a repetition of the validity check we recommend a consideration of the self-weight of not only the load cell but also of the rope, the rope tension system and the sensor unit itself. Alternatively, a different lightweight load cell would be more suitable.
- The reference load cell can measure loads up to 500 kN. The loads applied with this cell during testing were about 14 kN. The calibration document of this load cell revealed measurement errors of ±2 kN in this load range. Therefore, a load cell with less deviation for the expected load range and reduced maximum load capacity should be used.
- The results shown do not allow for an extensive error analysis as there are too few measurements. Testing should be performed sampling at different load steps for each load case.
- The thermal influence was neglected. A well-documented load-temperature dependency would help to interpret future measurements.
- The self-weight of the post has been neglected. This is acceptable if only load changes are of interest. However, it should be documented how much the mounting of the post influences the interaction between the steel plate and the four single sensors.
- The influence of possible bending in the steel plate which connects each load cell is not considered in the approach described above. Therefore, instead of a simple validity check of the measurements a full sensitivity analysis should be performed (see Section 7).

**Table 1:** Comparison between loads T and S applied through a fixed clamped post at its top and actions M and F on foundations calculated from sensor unit’s measurements.

Load Direction	“–u”		“+u”		Up		Down	
$T_{u}$ and $M_{u}$ in kNm	–4.2	–4.6	–10.3	–8.9	–54.3	–55.8	132	135.2
$T_{v}$ and $M_{v}$ in kNm	–57.8	–58.2	61.1	61.8	0.1	0.4	4.9	–1.9
$T_{w}$ and $M_{w}$ in kNm	–7.4	–6.1	7.0	6.8	–1.9	0.0	4.9	–0.4
$S_{u}$ and $F_{u}$ in kN	–13.2	–11.0	13.9	11.4	–0.1	–0.7	1.4	1.3
$S_{v}$ and $F_{v}$ in kN	1.0	0.4	2.4	2.3	12.1	10.3	–29	–25
$S_{w}$ and $F_{w}$ in kN	–0.1	0.4	–0.4	–1.1	4.3	4.3	–9.8	–10.6

## 7. Calibration

To calibrate a multi-axial sensor unit as described above, not only the sensitivity of the single channels, but also the influence of each single channel on all other channels has to be determined. This means sensitivity to tributary components resulting in a corresponding overall decoupling matrix. Beyeler *et al.* [12,13] describe a potentially suitable procedure for a micro-mechanical sensor device, which has been applied to the sensor unit presented in this section.

For direct calibration a conversion matrix K (see Equation (4)) has to be specially adapted to the sensor unit. The conversion matrix K has 6×12=72 unknown elements. Following this, several load cases must be applied with known loads ${\left[M\;F\right]}^{T}$ and the corresponding measurements $R_{\mathrm{raw}}$. For each load case, the conditions of equilibrium deliver six new equations. With a sufficient number of linearly independent equations, all unknowns can be determined. Due to standard measurement errors the entire system of equations should be solved using a least-square optimization method. This is especially the case because ideally more equations than unknowns are available. In this case the conversion matrix K is obtained by

(6) $$K_{6\times 12}={\left[\begin{array}{c} M\\ F \end{array}\right]}_{6\times 1}\cdot R_{\mathrm{raw}}^{T}\cdot {\left(R_{\mathrm{raw}}\cdot R_{\mathrm{raw}}^{T}\right)}^{-1}$$

The above calculation has to be performed for each single measurement throughout the measuring time series. It can also be achieved using spreadsheet software. In this case, ${\left[M\;F\right]}^{T}$ and $R_{\mathrm{raw}}^{T}$ are used with the values written in single columns and for each load case in single rows. This setup then also can be used for the post-processing of a dynamic measurement with several thousands time steps.

The above calibration procedure can produce a conversion matrix K that looks quite different to the one derived in Section 5.3. Table 2 compares the coefficients of K that we obtained according to Section 5.3 and Section 7. This comparison is the relationship between single coefficients of both matrices $K_{\mathrm{calibr}}$ and $K_{\mathrm{analyt}}$. A value 1 signifies identical coefficients but only 12 out of 72 coefficients are this similar. All others vary with different magnitudes up to a maximum of 10,505. These values show that single sensor channels have appreciable influence due to their cross sensitivity.

The determination of the conversion matrix K involves obtaining the 6 DOF loads for a given measurement. If therefore we perform a reverse calculation, Table 3 lists the differences between the target values for ${\left[M\;F\right]}^{T}$. In fact, the resulting actions ${\left[M\;F\right]}^{T}$ on the foundation do not differ too much. The deviations according to the procedure of Section 5.3 are larger than those of the calibration procedure. The latter apparently also take into account the interaction between the single load cells through the two steel plates. The deviations of the calibration procedure all stay below 1% of the measurement range indicating that the system of equations can be satisfactorily resolved for the application regarding the action on foundations of steel posts for rockfall protection systems.

**Table 2:** Conversion matrices K determined according to Section 5.3 and Section 7.

**Analytical derivation of K according to Section 5.3**											
6487	370	37,498	362	–5964	37,204	6	6104	–37,229	–6572	67	–37,468
–786	5362	–47,944	6533	–128	47,969	–5955	112	–48,037	822	–5942	48,004
12,594	9730	465	10,113	12,569	207	9938	12,895	268	12,715	9760	299
–30	38,611	2084	39,980	–18	–1479	–39,342	119	1205	57	–39,258	–1298
–39,196	–241	174	368	39,110	1795	–320	–40,203	–1770	39,594	–168	–79
–2432	–1335	–150,097	–1668	390	–149,893	–167	293	–149,978	–2530	167	–149,918
**Calibrated conversion matrix** K **according to Section 7**											
–37,127	–203,622	–395,156	67,306	–133,674	–10,946	–65,216	197,478	193,184	298,491	337,697	139,860
4030	31,124	–75,109	–45,454	16,703	83,877	178,938	21,951	–239,999	–3744	–75,575	–217,935
16,811	78,446	111,919	58,748	13,714	–30,916	–3301	–175,345	34,804	–171,443	–12,956	–34,459
4412	64,727	7304	28,010	10,370	7083	29,986	–16,368	–82,856	–26,101	–83,493	–118,425
–28,910	76,596	133,703	–13,902	83,959	–18,173	–4150	–138,244	–101,596	–77,949	–139,696	–92,887
–3581	27,968	–113,228	–1747	8313	–146,761	18,284	–28,923	–192,311	–38,164	–37,642	–191,804
**Comparison between analytical derivation and calibration of** K **(**$K_{\mathrm{ij},\mathrm{calibr}}/K_{\mathrm{ij},\mathrm{analyt}}$**)**											
–6	–550	–11	186	22	0	–10,505	32	–5	–45	5051	–4
–5	6	2	–7	–130	2	–30	196	5	–5	13	–5
1	8	241	6	1	–149	0	–14	130	–13	–1	–115
–146	2	4	1	–567	–5	–1	–137	–69	–458	2	91
1	–318	767	–38	2	–10	13	3	57	–2	832	1182
1	–21	1	1	21	1	–109	–99	1	15	–226	1

The calibration scheme should be setup and documented carefully, e.g., to consider also process- induced influences such as the self-weights of the mounted steel post and the load cells. In the case of Table 2, the loads applied were recorded with a 35 kN load cell and care was taken to ensure that the single load cases, *i.e.*, the directions in which and the positions where the load is applied, are linearly independent. Otherwise, the above system of equations will be ill-conditioned. If the circumstances in the field are not optimal and load cases are linearly dependent, the above system of equations still provides a resulting conversion matrix K which, however, might look completely different as shown for example in Table 4.

**Table 3:** Deviation of calculated actions on foundation ${\left[M\;F\right]}^{T}=K\cdot R_{\mathrm{raw}}$ based on fourteen load cases determined according to Section 5.3 and Section 7.

**Differences between applied actions on foundation and calculated using an analytically derived conversion matrix K**														
2.3	0.7	2.0	–0.1	3.5	3.4	6.5	0.2	–3.4	–1.1	3.8	4.9	3.5	4.1	kNm
–0.1	–0.2	0.7	–0.2	–0.8	–1.0	–0.1	–1.4	0.1	0.9	1.7	1.0	–2.8	–0.5	kNm
0.0	0.0	1.0	–0.1	–3.6	–5.5	–0.6	–4.6	0.0	1.4	2.6	0.9	–0.4	–1.0	kNm
–0.2	–0.4	–0.4	–0.5	–0.1	0.0	–0.1	–0.1	–0.2	0.0	–0.1	0.1	–0.9	0.3	kN
–1.3	–1.6	–1.3	–1.7	–1.7	–1.7	–1.3	–1.1	–0.8	–0.8	–0.9	–1.1	–0.7	–1.0	kN
–0.2	0.1	–0.2	0.2	0.0	0.0	–0.3	0.1	–0.2	–0.4	–0.5	–0.4	–0.4	–0.3	kN
**Differences between applied actions on foundation and calculated using a calibrated conversion matrix** K														
0.9	–0.3	1.0	–0.7	0.2	–0.6	0.8	–0.4	1.0	–0.8	–0.8	1.1	–0.1	–0.4	kNm
0.6	–0.2	0.0	0.0	–0.3	0.4	0.0	0.0	–0.1	0.0	–0.3	0.4	–1.1	0.8	kNm
0.1	0.1	–0.1	0.0	0.0	0.0	0.0	0.0	0.0	0.0	0.0	0.0	0.0	0.0	kNm
0.3	–0.1	0.0	0.0	–0.1	0.2	0.0	0.0	0.0	0.0	–0.1	0.2	–0.5	0.3	kN
–0.3	0.0	–0.2	0.2	0.0	0.1	–0.2	0.1	–0.2	0.2	0.2	–0.3	0.1	0.1	kN
0.0	0.0	–0.1	0.1	0.0	0.1	–0.1	0.0	–0.1	0.1	0.1	–0.1	0.0	0.1	kN

**Table 4:** Conversion matrices K determined according to Section 7 resulting from an inadequate calibration process.

9.9 × 10${}^{6}$	–1.2 × 10${}^{7}$	–5.3 × 10${}^{6}$	–1.2 × 10${}^{7}$	–1.2 × 10${}^{7}$	2.5 × 10${}^{6}$	1.2 × 10${}^{7}$	1.1 × 10${}^{7}$	–1.8 × 10${}^{6}$	–1.1 × 10${}^{7}$	9.4 × 10${}^{6}$	4.0 × 10${}^{6}$
–5.8 × 10${}^{7}$	1.0 × 10${}^{8}$	4.2 × 10${}^{7}$	1.1 × 10${}^{8}$	8.7 × 10${}^{7}$	–3.0 × 10${}^{7}$	–1.0 × 10${}^{8}$	–6.8 × 10${}^{7}$	2.3 × 10${}^{7}$	7.1 × 10${}^{7}$	–7.9 × 10${}^{7}$	–2.7 × 10${}^{7}$
–7.1 × 10${}^{6}$	1.2 × 10${}^{7}$	5.2 × 10${}^{6}$	1.3 × 10${}^{7}$	1.1 × 10${}^{7}$	–3.7 × 10${}^{6}$	–1.3 × 10${}^{7}$	–8.3 × 10${}^{6}$	2.8 × 10${}^{6}$	8.6 × 10${}^{6}$	–9.6 × 10${}^{6}$	–3.4 × 10${}^{6}$
–1.3 × 10${}^{7}$	2.3 × 10${}^{7}$	9.7 × 10${}^{6}$	2.4 × 10${}^{7}$	2.0 × 10${}^{7}$	–6.9 × 10${}^{6}$	–2.4 × 10${}^{7}$	–1.6 × 10${}^{7}$	5.3 × 10${}^{6}$	1.6 × 10${}^{7}$	–1.8 × 10${}^{7}$	–6.3 × 10${}^{6}$
–2.1 × 10${}^{6}$	2.5 × 10${}^{6}$	1.1 × 10${}^{6}$	2.5 × 10${}^{6}$	2.6 × 10${}^{6}$	–5.3 × 10${}^{5}$	–2.5 × 10${}^{6}$	–2.3 × 10${}^{6}$	4.0 × 10${}^{5}$	2.3 × 10${}^{6}$	–2.0 × 10${}^{6}$	–8.2 × 10${}^{5}$
–2.2 × 10${}^{6}$	2.6 × 10${}^{6}$	1.1 × 10${}^{6}$	2.6 × 10${}^{6}$	2.9 × 10${}^{6}$	–6.7 × 10${}^{5}$	–2.6 × 10${}^{6}$	–2.4 × 10${}^{6}$	2.2 × 10${}^{5}$	2.5 × 10${}^{6}$	–2.0 × 10${}^{6}$	–1.0 × 10${}^{6}$

## 8. Conclusions

Knowing the loads of a steel post within a rockfall protection barrier during a rockfall event is an important task. The post’s foundation must be capable of transferring the dynamic loads safely and stably to the ground. In the presented case, the foundation of the post is mounted on a near vertical rock face 15 m above the ground. A newly developed measuring device is presented that is capable of detecting torques and forces, each in three orthogonal directions. The challenges for the design of such a system were the magnitude of the expected loads with up to 1.2 MN parallel to the steel post in addition to the high ground-parallel forces, as well as high torque loads due to posts fix-clamped to the ground. Furthermore, the device is installed outside throughout the year and therefore requires a corresponding robustness. A system consisting of four triaxial force sensors between two massive steel plates was selected, which in total records twelve force channels.

The measurements have to be converted into the six torques and forces acting on the foundation. To enable this the conversion scheme is based on the calibration matrices of the single force sensors and on the geometrical setup of the sensor unit. This scheme represents the main loads acting on the sensor unit reasonably well. However, the loads orthogonal to a main load direction diverge from the expected results when compared to loads of precisely known values. A better conversion from measurements to loads on the foundation is therefore presented and requires a calibration of a 6×12 conversion matrix. For an *in situ* calibration, the sensor unit remains installed in the field. A steel post is clamped to the sensor unit’s top surface and loaded with at least twelve different load cases. These load cases are produced by attaching a steel cable between the post and different anchorage points under tension. The tension in the cable, its direction and the point of load application on the post are recorded in parallel to quasi-statically recording the loads in the four triaxial sensors. All measurements provide the input data for an system of equations that determines the conversion matrix from the load measurements to the actions on the foundation. The system of equations is solved using a least square optimization.

If a sensor’s channel fails the calculation procedure from measurements to the loads acting on the foundation will work as long as more than 6 channels are still measured. However, in this case a re-calibration of the system without the failed channel is necessary.

It also has to be considered that for a full calibration and to quantify the linearity of the measurements according to [26,27] the setup described above has to be enhanced for several load steps within each load case. The German Calibration Service [27] provides different possible calibration procedures. For example, one procedure requires three times pre-loading to the maximum load and then twice a step-wise loading. Such a procedure allows the determination of intrinsic measurement errors. Regarding the sensor unit presented in this contribution, a corresponding procedure allowing the quantification of a standard measurement error has still to be developed. However, the sensor unit delivers usable measurements for a rigorous design of the post’s foundation. A number of technical issues have been highlighted that will improve the precision of the conversion matrix presented. Finally, we recommend that the influence of the air temperature and solar radiation on the measurements is studied. However, if only very short-term measurements during a rockfall test are of interest, thermal influences can be neglected.
